# How can Blood Flow Restriction Exercise be Utilised for the Management of Persistent Pain Following Complex Injuries in Military Personnel? A Narrative Review

**DOI:** 10.1186/s40798-024-00804-7

**Published:** 2025-02-04

**Authors:** Luke Gray, Peter Ladlow, Russell J Coppack, Robyn P Cassidy, Lynn Kelly, Sarah Lewis, Nick Caplan, Robert Barker-Davies, Alexander N Bennett, Luke Hughes

**Affiliations:** 1 Department of Sport, Exercise and Rehabilitation, Northumbria University, Newcastle, United Kingdom; 2 Academic Department of Military Rehabilitation, Defence Medical Rehabilitation Centre – Stanford Hall, Loughborough, United Kingdom; 3 Department for Health, University of Bath, Bath, United Kingdom; 4 Defence Medical Rehabilitation Centre – Stanford Hall, Loughborough, United Kingdom; 5 School of Sport, Exercise, and Health Sciences, Loughborough University, Loughborough, United Kingdom; 6 Faculty of Medicine, National Heart and Lung Institute, Imperial College London, London, United Kingdom

**Keywords:** Blood flow restriction, Military, Musculoskeletal injury, Persistent pain, Rehabilitation

## Abstract

**Background:**

Persistent pain is a complicated phenomenon associated with a wide array of complex pathologies and conditions (e.g., complex regional pain syndrome, non-freezing cold injury), leading to extensive disability and reduced physical function. Conventional resistance training is commonly contraindicated in load compromised and/or persistent pain populations, compromising rehabilitation progression and potentially leading to extensive pharmacological intervention, invasive procedures, and reduced occupational status. The management of persistent pain and utility of adjunct therapies has become a clinical and research priority within numerous healthcare settings, including defence medical services.

**Main Body:**

Blood flow restriction (BFR) exercise has demonstrated beneficial morphological and physiological adaptions in load-compromised populations, as well as being able to elicit acute hypoalgesia. The aims of this narrative review are to: (1) explore the use of BFR exercise to elicit hypoalgesia; (2) briefly review the mechanisms of BFR-induced hypoalgesia; (3) discuss potential implications and applications of BFR during the rehabilitation of complex conditions where persistent pain is the primary limiting factor to progress, within defence rehabilitation healthcare settings. The review found BFR application is a feasible intervention across numerous load-compromised clinical populations (e.g., post-surgical, post-traumatic osteoarthritis), and there is mechanistic rationale for use in persistent pain pathologies. Utilisation may also be pleiotropic in nature by ameliorating pathological changes while also modulating pain response. Numerous application methods (e.g., with aerobic exercise, passive application, or resistance training) allow practitioners to cater for specific limitations (e.g., passive, or contralateral application with kinesiophobia) in clinical populations. Additionally, the low-mechanical load nature of BFR exercise may allow for high-frequency use within residential military rehabilitation, providing a platform for conventional resistance training thereafter.

**Conclusion:**

Future research needs to examine the differences in pain modulation between persistent pain and pain-free populations with BFR application, supporting the investigation of mechanisms for BFR-induced hypoalgesia, the dose-response relationship between BFR-exercise and pain modulation, and the efficacy and effectiveness of BFR application in complex musculoskeletal and persistent pain populations.

## Key Points

• Conventional resistance training (e.g., > 70% 1RM) is commonly contraindicated in load compromised and/or persistent pain populations, delaying rehabilitation and reducing military operational readiness.

• Blood flow restriction application could alleviate pain following complex injury or persistent pain in military settings, reducing the burden on the individual and UK Defence Medical Services.

• The plethora of blood flow restriction applications provide sport and exercise medicine practitioners with many options based on their specific aims and objectives during rehabilitation.

## Background

Persistent pain, defined as pain lasting > 3 months [1], affects between one-third and one-half of UK residents [2], with 10.4–13.3% reporting moderate-to-severe disabling pain [3]. The combined economic cost for persistent pain pathologies within the UK is unknown; however, low back pain and osteoarthritis, together, costs the National Health Service >£15 billion annually [4, 5]. From a patient perspective, persistent pain can significantly reduce an individual’s quality of life through increased disability and reduced physical function [6]. Subsequently, this may adversely affect personal finances through reduced productivity and more time off work [7] negatively influencing emotional wellbeing [8]. Within the literature, it is commonly discussed whether pain should be its own disease entity [9]; however, it is accepted as being a complex phenomenon, with persistent pain development involving abnormal somatosensory processing [10]. Conversely, a vast array of persistent and complex conditions, have their own individual pathophysiology which can result in persistent pain, and occur following acute traumatic injury, surgery, or innocuous event, resulting in lifechanging and prolonged disability, loss of function, and pain, with poor long-term prognosis [11–13].

Commonly within rehabilitation settings, conventional resistance training (> 70% 1RM) is utilised to promote positive physiological adaption following musculoskeletal injury [14, 15]. Although heavy resistance training has been shown to reduce pain in persistent pain populations [16], the stimulus required to evoke a beneficial exercise-induced hypoalgesic response requires high-intensity short-duration (e.g., traditional aerobic or resistance training) and/or low-intensity long-duration exercise (e.g., submaximal isometric contractions) [17–19]; however, this is often unachievable and unfeasible in load compromised and/or persistent pain populations due to self-immobilisation and symptomatic impairments [20, 21]. Unfortunately, there are few other exercise rehabilitation interventions that can attenuate the pain response following complex musculoskeletal and/or neurological injury, leading to the widespread use of pharmacological intervention, including opioids, which can have profound negative implications on health [22].

Blood flow restriction (BFR) exercise involves inflation of a pneumatic tourniquet to apply external pressure around the most proximal aspect of an appendicular upper or lower limb [23], resulting in compression of the underlying vasculature, creating a hypoxic environment through partial and complete arterial and venous occlusion, respectively [24, 25]. Research has shown that BFR exercise can elicit greater morphological and physiological adaptions to skeletal muscle compared to conventional aerobic and resistance exercise with equal mechanical/external load [23, 26–29], while also provoking substantial hypoalgesia [30, 31]; thus, offering profound utility in load compromised and persistent pain populations [32–36].

Persistent pain associated with complex musculoskeletal and/or neurological injury is common within military populations [37]. Currently, musculoskeletal injury is a leading cause for medical downgrading within the British Armed Forces, and persistent pain is a significant contributing factor in a number of these cases [38]. Persistent pain has been reported as a leading cause of disability within military personnel, reducing operational readiness [39]. Reif et al. [40] reported ~ 63% of active US service members had at least one pain diagnosis, with over 50% being of musculoskeletal origin. Furthermore, 44% of US service personnel returning from combat deployment reported persistent pain [41]. Additionally, in 2022–2023, the British Armed Forces had 1576 medical discharges at a rate of 30 per 1000 personnel, with 26%, 37% and 46% of discharges relating to musculoskeletal disorders and injuries for the Royal Air Force, British Army and Royal Navy, respectively [42]. Furthermore, a large proportion of these medical discharges were for musculoskeletal injury or disorder of the lower limb [42]. Within a cohort of the ADVANCE study [43], Vollert et al. [44] reported that at a median of 8-years since injury or deployment, 46% of individuals reported mild (1–3 numerical pain rating scale [NPRS]), 15% moderate (4–7 NPRS) and 6% extreme (> 8 NPRS) pain. Given the contextual factors surrounding pain as an entity, and the complexity of pain diagnosis, or multiple diagnosis, and length of symptoms, the true burden of pain is still unclear and complex intervention may be a necessity to reduce the impact on operational readiness [40]. Therefore, any adjunct therapy or intervention that accelerates the progression of rehabilitation by modulating pain is of interest not only to the rehabilitation and sport and exercise medicine communities, but also the wider community health services. The aim of this narrative review, therefore, is to explore the use of BFR exercise to elicit hypoalgesia, the mechanisms of effect, and the potential implications this may have during rehabilitation in complex conditions where persistent pain is the primary limiting factor to progress, within defence rehabilitation healthcare settings.

## Main Text

### Blood Flow Restriction Application

The application of BFR can occur during voluntary exercise (i.e., aerobic or resistance training), passive activity (i.e., rest) or complementary therapies (e.g., neuromuscular electrical stimulation [23]. Haemodynamic [45], neuromuscular [46, 47], and perceptual [28, 48] responses to BFR can be manipulated through relative pressure application. Aerobic and resistance exercise with BFR should be completed with partial arterial occlusion, whereas passive application has complete arterial occlusion [23, 49]. Please refer to Table 1 for an overview of general guidelines for BFR application. Readers are directed to Patterson et al. [23] for an in-depth review of BFR application and methodology.

### Effects of Blood Flow Restriction Exercise on Pain

In recent years, despite BFR-induced hypoalgesia research being in its infancy, the literature has significantly grown, and suggests BFR exercise can be a viable option to ameliorate pain across numerous clinical populations [50–54]. Research currently suggests that a dose-response relationship exists, with a greater hypoalgesic response being seen following BFR exercise using higher limb occlusion pressures (80% vs. 40%), in both aerobic and resistance training [30, 31]. Furthermore, Yang et al. [55] reported significant hypoalgesia when utilising low-load moderate pressure (60% limb occlusion pressure) BFR resistance exercise at 30% 1RM to failure, when compared to moderate conventional RT loads (50% 1RM). Consensus on the optimal exercise frequency, intensity and volume is, however, still lacking due to significant heterogeneity within the published literature [35, 56]. Briefly, the hypoalgesic response following BFR exercise is thought to occur from a combination of mechanisms, including activation of opioid and endocannabinoid systems, cardiovascular and pain regulatory system communication, conditioned pain modulation, high threshold motor unit recruitment, and other potential local mechanisms [57–59]. Conversely, there are still unknowns regarding the physiological mechanisms of BFR-induced hypoalgesia [36, 60]. For more in-depth reviews regarding the mechanisms of BFR-induced hypoalgesia, please see Cervini et al. [59], Hughes and Patterson [57], and Song et al. [58].

### Current Rationale for BFR Application in Persistent Pain and Military Rehabilitation

The Defence Medical Rehabilitation Centre (DMRC) at Stanford Hall offers a consultant-led, multi-disciplinary approach to intensive residential rehabilitation, with specialists being utilised across dietetics, mental health, occupational therapy, pain management, podiatry, prosthesis/orthosis provision, social work, and speech and language, to support throughout, creating a unique rehabilitation environment [61], that has shown efficacy for improving clinical outcomes in a wide array of musculoskeletal injuries [54, 62, 63]. Additionally, the feasibility and acceptability of BFR exercise within UK Defence Rehabilitation has already been established [54, 64], with additional research ongoing to establish the efficacy of BFR in the management of persistent knee pain within a residential setting [65].

The lower mechanical demands of BFR resistance training allow earlier implementation following musculoskeletal injury, and at a greater training frequency, thus accounting for early skeletal muscle hypertrophy [23]. Efficacy over longer periods (> 8 weeks) has also been demonstrated within the literature [66, 67]. Significant neuromuscular adaptions are produced following short-term, high frequency (> 4 days/week, < 3 weeks) BFR exercise, indicative of potential use within in-patient and residential settings [68]. Additionally, blocks of high-frequency BFR resistance training may also allow greater levels of skeletal muscle hypertrophy to be attained in subsequent periods of conventional resistance training, relating to increased satellite cell proliferation and myonuclear accretion [69]. Literature has also indicated that increases in muscle strength and mass occur directly proximal to the occlusion cuff site, in upper and lower limb musculature [70–73]. It is theorised that the increased activation of proximal musculature due to distal fatigue, and systemic anabolic signalling, are potential mechanisms for proximal adaption [70, 71], although the physiological mechanisms are still somewhat speculative [74]. Nevertheless, these findings provide significant benefit following hip arthroscopy and anterior cruciate ligament reconstruction, where proximal musculature strengthening, and control are key for successful post-operative outcomes [75, 76]. Additionally, following independent, unilateral upper- and lower-limb BFR exercise, contralateral increases in muscular strength and task-related performance have been reported, indicating a systemic, crossover mechanism [71, 77–80]. Further mechanistic research into contralateral and proximal adaption following BFR exercise is warranted, although the existing research offers an opportunity for remote adaptation in complex clinical cases [74, 81]. Together, this culminates in the potential for extensive BFR applications within residential defence rehabilitation settings.

### Persistent Pain in Military Populations

A vast array of persistent pain and complex musculoskeletal and/or neurological pathologies exist within military populations [82, 83]. For this review, specific pathologies of central interest to UK Defence Rehabilitation, including complex regional pain syndrome, non-freezing cold injury, persistent post-operative pain, and post-traumatic osteoarthritis are discussed [84]. These pathologies have been selected due to the challenges associated with their rehabilitation due to pain limiting various exercise rehabilitation variables (e.g., exercise selection, loading strategies, and volume of training), thereby limiting progression. The scope and potential role of BFR exercise acting as a primary driver and modulator of pain for the management of these complex conditions are explored.

#### Complex Regional Pain Syndrome

Presenting with pain disproportionate to the injury or inciting event, complex regional pain syndrome (CRPS) patients also present motor/tropic, sudomotor and vasomotor responses [85], resulting from local and systemic, immunological, inflammatory, and neurological changes [85–87]. Ankle dislocations or intra-articular fractures, high impact trauma, high levels of pain after trauma, limb immobilization and prior post-traumatic stress disorder are all known risk factors for CRPS development [85, 88, 89]. Furthermore, occupational hazards relating to combat significantly increase CRPS incidence [90]. Complex regional pain syndrome prognosis varies, with studies suggesting up to 74% symptom remission within 1-year and 36% after 6-years [91, 92]. Thirty-one percent of individuals, however, describe permanent inability to return to work, indicative of a high social and psychological burden [93, 94]. Locally, at DMRC, several patients are in the early stages of CRPS development, and do not reach the Budapest Criteria threshold for diagnosis [95]; however, they typically present with a range of CRPS-associated symptoms (e.g., allodynia, hyperalgesia, motor dysfunction, oedema), and thus are termed CRPS-like patients, and referred to the appropriate interdisciplinary services available with UK Defence Rehabilitation. Despite published international guidelines, optimal treatment of CRPS is unknown [96–98]. Nevertheless, treatment is often multi-factorial and multi-disciplinary, with a shared focus on pain management [98, 99]. Once extensive conservative care (e.g., exercise, pharmacology), and invasive procedures such as spinal cord stimulation, have been exhausted and to no avail, amputation is considered the last resort [100–102]. Research suggests limb amputation can increase mobility, improve quality of life, and reduce persistent pain in CRPS patients [103]. However, significant negative sequelae including recurrence, phantom limb pain, and psychological distress can occur [104], with a significant increase in all-cause mortality within 5-years following amputation [105]. With research suggestive of poor prognosis [94], extensive pharmacological management [102] and limb amputation being a viable but significant treatment option [103], the use of a novel non-invasive therapeutic exercise intervention shown to alleviate persistent pain earlier in the clinical pathway could be considered [35].

To our knowledge, there is only one published case study investigating the use of BFR in CRPS patients [12]. Ridenhour [12] theorized that microvascular, muscular and oxygenation adaptions promoted by three unspecified, lower limb BFR resistance training sessions were responsible for the reduction in pain and increased function. However, three sessions are likely insufficient to promote significant aerobic, microvascular, or morphological adaption [23, 29, 106], thus suggesting other mechanisms may have significant influence. Conditioned pain modulation, a proposed mechanism of BFR-induced hypoalgesia [57, 58], is commonly utilised within CRPS patients through pain exposure therapy and spinal cord stimulation, thus showing a viable mechanistic rationale [107, 108]. As kinesiophobia is common in CRPS patients [109], passive BFR could be utilised to elicit hypoalgesia without movement by potentially utilising conditioned pain modulation [49, 110]. Additionally, pain exposure physical therapy has gained traction within the literature, with beneficial outcomes being reported [111–113]. As BFR exercise is often perceived as painful (discomfort to the exercising muscle), passive BFR application could help desensitise the symptomatic region, as well as improve cardiac autonomic response, increased corticomotor excitability, and promote positive endothelial adaption [114–117], which target specific CRPS pathophysiological changes [118–120], thus aiding in symptomatic relief and disease regression.

#### Non-Freezing Cold Injury

Commonly affecting hands and feet, non-freezing cold injuries (NFCI) present with debilitating persistent pain, hyperhidrosis, and cold sensitivity, causing significant economic and operational burden to the UK Ministry of Defence [11]. Stemming from prolonged exposure to wet and cold environments, the pathophysiology of NFCI is still largely misunderstood; however, ischemia-reperfusion injury is plausible [11, 121]. Following prolonged cold exposure, it is rationalised that cyclic, poorly regulated, microcirculatory vasoconstriction and vasodilation occurs leading to ischemia-reperfusion injury [122]. Localised hypoxia promotes significant reactive oxygen species production, in turn increasing cellular oxidative stress and DNA damage [123, 124]. The localised proinflammatory environment promotes mitochondrial dysfunction, and thus cell apoptosis and peripheral nerve damage, as well as further increasing tissue vulnerability during reperfusion moments [125, 126]. Subsequently, muscular contractile dysfunction, necrosis and oedema are more prevalent during reperfusion relating to endothelial dysfunction, lactic acidosis, and rhabdomyolysis [127–129]. Additionally, reperfusion further increases reactive oxygen species production, resulting in further negative microenvironment changes [122, 130]. Occupational requirements of military activities result in greater exposure to cold environments compared to most civilian populations, thus increasing the risk of NFCI in military populations [121, 131, 132]. As a dearth of treatment modalities exist, there is an emphasis on NFCI prevention strategies [121, 133]. Significant emphasis should be placed on mitigating persistent irretractable pain through early intervention with amitriptyline after ischemia-reperfusion injury [132]. Additionally, secondary sequelae may occur following NFCI [134], such as ulceration and tissue loss, which can develop in the severe neuropathic foot, leading to minor or major limb amputation [135]. As treatment options are limited [121, 133], the use of any novel and non-invasive therapeutic exercise intervention shown to alleviate persistent pain could be considered [35].

Ischemic preconditioning, or passive BFR application, offers a protective effect against further ischemic muscle injury following a period of acute ischemia in chronic lower-limb ischemic patients [136] through increased collateral blood flow and levels of local inflammatory mediators [136, 137]. Additionally, BFR exercise also promotes angiogenesis, to a greater degree than non-occluded exercise, and reduces mitochondrial reactive oxygen species production, both factors for ischemia-reperfusion injuries [138–140]. Additionally, the transient hypoxic environment created by BFR exercise, along with increased shear stress during reperfusion, improves endothelial function [117], which may help address the endothelial dysfunction commonly seen in NFCI [125, 126]. Moreover, BFR exercise has been established as promoting beneficial adaption in human immune deficiency virus infection, kidney disease, rheumatoid arthritis, and type 1 and 2 diabetes mellitus patients where peripheral neuropathy is a commonly associated pathology [141–149]. Therefore, BFR application could ameliorate and promote beneficial physiological adaptions associated with NFCI, as well as providing acute hypoalgesia [30, 31].

#### Persistent Post-Operative Pain

During an 11-year period up to 2014, > 2800 surgeries were performed on appendicular limbs within serving UK military personnel [150]. Concerningly, post-operative persistent pain can affect up to 50% of patients [151, 152], resulting in significant functional limitations, psychological trauma, and considerable economic and operational burden to the UK Ministry of Defence [153]. Post-operative persistent pain is complicated, with factors such as complexity of surgery (e.g., arthroscopic vs. open), duration of surgery and tourniquet time, immune function, patient age, and pre-operative pain influencing inflammation and nerve-related injury, and consequently peripheral and central pain sensitisation [154–156]. Pre-operative surgical site pain, opioid use, and post-operative high-intensity pain lasting > 5 days are consistently reported as risk factors for persistent pain development within the literature [152, 155]. Primary treatment consists of long-term pharmacological management strategies, including gabapentin, ketamine, and opioids, that have widespread negative side effects [152, 157]. When primary pharmacological management fails, invasive procedures such as botulinum toxin and nerve block injections, nerve ablation, short-term implantation of neuromodulation device, and further surgical intervention are considered [153]. With subsequent invasive procedures and pharmacological management being primary and secondary options [152, 153, 157], the use of a non-invasive or pharmacological management strategy that can attenuate pain and improve physical function, such as BFR exercise, should be considered [57, 153].

Post-operative use of BFR training is well established within musculoskeletal injury rehabilitation literature [32] and is shown to reduce post-operative pain levels during early recovery, with the intervention safely utilised from day 0 post-operatively [158], offering a favourable platform for future rehabilitation [34, 159]. Statistically significant post-operative reductions in pain following BFR intervention have been recorded after anterior cruciate ligament reconstructions, distal radius fractures, and tibial osteotomies, indicating widespread utility [51, 160, 161]. Persistent post-operative pain research suggested elevated levels of interleukin-6, and inhibition of mammalian target of rapamycin and mitogen activated protein kinase signalling may contribute towards chronicity at a cellular level [162]. Conversely, BFR exercise has been shown to promote activation of mitogen activated protein kinase and mammalian target of rapamycin pathways [163]. Additionally, Nielsen et al. [164] reported statistically significant reductions in interleukin-6 3-h post-BFR exercise, indicating BFR application may influence homeostasis. However, this result is inconsistent within the literature, likely due to the vast heterogeneity in BFR exercise prescription [165]. Through leveraging the conditioned pain modulation effect of passive BFR pre-operatively [110, 166], the application may be pleiotropic, with reductions in post-operative circulating tumour necrosis factor alpha also found following pre-operative bouts of occlusion [167]. Furthermore, near-immediate post-operative application could elicit favourable reductions in pain without the need for movement and mitigate risk factors for chronicity [49, 155, 168].

#### Post-Traumatic Osteoarthritis

Commonly affecting younger, active populations, post-traumatic osteoarthritis (PTOA) results in significant pain and considerable functional disability [169, 170]. Responsible for up to 12% of all osteoarthritis cases, PTOA occurs secondary to a traumatic injury often associated with athletic or military activities [171], leading to downgrading of duties and/or medical discharge [38]. Post-traumatic osteoarthritis typically occurs in the lower limbs (e.g., ankle, hip, and knee joints) following intra-articular fracture with or without subchondral bone disruption, or joint dislocations with associated ligamentous injury [172]. It may also affect the glenohumeral joint following frequent instability episodes [173]. The progressive disease pathogenesis includes chondrocyte apoptosis, inflammation and cytokine release in the cartilage and synovium, mitochondrial dysfunction, reactive oxygen species production, and subchondral bone remodelling [174–176]. Additionally, the inflammation associated with the pathogenesis of PTOA is often considered a driver for pain response following joint injury [177]. Consequently, to date, there are no disease-modifying medical therapies for PTOA [170], with symptom management being the primary focus through education, exercise rehabilitation, and pharmacological management [178], with surgical intervention considered on an individual basis due to an increased risk of post-surgical complications including acute deep vein thrombosis, further revisions required, infection, and stiffness [179]. As treatment to date focuses on symptom management [178], a novel therapeutic exercise intervention to alleviate persistent pain and increase physical function is warranted [35, 36].

A plethora of literature exists investigating the effects of BFR exercise on osteoarthritis, with a reduction in symptoms and increased function being commonly reported [36, 52, 180–182]. As part of PTOA pathophysiology, the local microenvironment experiences changes to numerous metabolic pathways, including tumour necrosis factor alpha and interleukin-6 [183, 184], leading to catabolic enzyme expression, downregulation of cartilage extracellular matrix synthesis and increased apoptotic pathway activity and, therefore, joint degeneration [185]. Furthermore, arthrogenic muscle inhibition is common sequelae of osteoarthritis, leading to muscle weakness, and reduced rehabilitation potential [186], relating to abnormal neural input (e.g., reduced motor neuron excitability, and central activation failure) arising from damaged mechanoreceptors, with additive localised inflammation also impairing mechanoreceptor and neural connections [187]. Emerging evidence suggests BFR exercise is effective at ameliorating arthrogenic muscle inhibition in post-operative military personnel [188], and is commonly used within anterior cruciate ligament reconstruction rehabilitation [189] whereby arthrogenic muscle inhibition is a common consequence of surgery [190]. Additionally, research suggests BFR exercise increases corticomotor excitability [116], and as previously mentioned, promotes homeostasis via reductions in circulating tumour necrosis factor alpha and interleukin-6 levels, thus influencing inflammation [191, 192]. BFR application could, therefore, be pleotropic in nature, by reducing inflammation therefore mediating pain generation, and attenuating other negative pathophysiological changes associated with PTOA, whilst also providing acute BFR-induced hypoalgesia [30, 31, 191, 192].

**Table 1:** Recommended General Prescription of BFR Application [23, 69, 74]

	*BFR-RT*	*BFR-AE*	***P***-*BFR*
Frequency	1-2x per day (1–3 weeks), 2-3x per week (> 3 weeks)	1-2x per day (1–3 weeks), 2-3x per week (> 3 weeks)	1-2x per day
Duration	> 8 weeks	> 8 weeks	6–8 weeks
Load	20–40% 1RM or 7–8 RPE	< 50% VO_2_max or HRR	N/A (supine & static)
Occlusion Duration	5–10 min per exercise	5–30 min per exercise	5 min per set
Sets & Reps	30-15-15-15, or sets to failure, 30s rest between sets	Continuous or interval based	3–5 sets, 2–5 min reperfusion between sets
Pressure	40–80% AOP	40–80% AOP	70–100% AOP

*AOP*, Arterial Occlusion Pressure; *BFR-AE*, Blood Flow Restriction with Aerobic Exercise; *BFR-RF*, Blood Flow Restriction with Resistance Training; *HRR*, Heart Rate Reserve; *P-BFR*, Passive Blood Flow Restriction; *RM*, Repetition Maximum; *RPE*, Rate of Perceived Exertion; VO_*2*_*max*, Maximum Rate of Oxygen Consumption

## Conclusion

To date, a limited amount of research exists investigating the influence of BFR applications on persistent pain, and on complex musculoskeletal pathologies. Furthermore, there is a limited number of registered randomised controlled trials publicly listed (clinicaltrials.gov). Future research should focus on examining the differences in pain modulation between persistent pain and pain-free populations with BFR application, which may allude to greater understanding of the mechanisms of BFR-induced hypoalgesia. Additionally, significant heterogeneity in BFR exercise prescription for hypoalgesia exists; thus, investigating dose-response relationships should be a focus to elucidate best practice and optimal delivery guidelines.

However, the application of BFR exercise across residential military rehabilitation has scope to include numerous persistent pain and complex musculoskeletal and/or neurological pathologies, with considerable mechanistic rationale for its use. The numerous methods of applying BFR (e.g., aerobic exercise, passive application, or resistance training) allow sport and exercise medicine practitioners to provide interventions taking into account condition-specific limitations (e.g., contralateral resistance training for single-limb kinesiophobia, ipsilateral resistance training when load-compromised or passive ipsilateral post-operatively). In addition, BFR exercise offers a multi-faceted approach to rehabilitation; both positive morphological and physiological adaptions are reported, as well as a modulation of pain, thus directly mitigating pathological changes and reducing sequelae. Furthermore, the low-mechanical load nature of BFR exercise may allow for high-frequency use within residential military rehabilitation, providing a platform for conventional resistance training thereafter. To conclude, BFR exercise and application offers a viable alternative to conventional rehabilitation methods and potentially pharmacological pain management strategies for persistent pain and complex musculoskeletal and/or neurological pathologies.

## Data Availability

Not applicable.

## Abbreviations

BFR: Blood flow restriction
CRPS: Complex regional pain syndrome
NFCI: Non-frozen cold injury
PTOA: Post-traumatic osteoarthritis
